# Long‐term peripheral immune cell profiling reveals further targets of oral cladribine in MS

**DOI:** 10.1002/acn3.51206

**Published:** 2020-10-01

**Authors:** Tobias Moser, Kerstin Schwenker, Michael Seiberl, Julia Feige, Katja Akgün, Elisabeth Haschke‐Becher, Tjalf Ziemssen, Johann Sellner

**Affiliations:** ^1^ Department of Neurology Christian Doppler Medical Center Paracelsus Medical University Salzburg Austria; ^2^ Department of Neurology Multiple Sclerosis Center, Center of Clinical Neuroscience Carl Gustav Carus University Hospital Technische Universität Dresden Dresden Germany; ^3^ Department of Laboratory Medicine Paracelsus Medical University Salzburg Austria; ^4^ Department of Neurology Klinikum rechts der Isar Technische Universität München München Germany; ^5^ Department of Neurology Landesklinikum Mistelbach‐Gänserndorf Mistelbach Austria

## Abstract

**Objectives:**

To expand the knowledge about the immunological consequences of cladribine (CLAD), a pulsed immune reconstitution therapy approved for active multiple sclerosis (MS), beyond the known short‐term effects on peripheral immune cell subsets.

**Methods:**

In this study, we characterized depletion and restitution kinetics as well as cytokine profiles of peripheral immune cell subsets in 18 patients with MS following treatment with oral CLAD. The methods involved blood collection prior to CLAD and every three months over a period of 24 months, and extensive characterization of various immune cells subsets by multiparametric flow cytometry.

**Results:**

We found a selectivity of CLAD towards central memory T cells and memory B cells and detected a hyper‐repopulation of maturing B cells. Counts of classical (−65%) and various nonclassical TH17 cells (−84% to −87%) were markedly reduced 24 months after treatment start, and were comparable with depletion rates of class‐switched memory B‐cell phenotypes (−87% to −95%). The nadir of TH cells was more pronounced in the second treatment year. We observed a proportional surge of CD20 T‐cell subsets and an expansion of regulatory T, B and NK cells. Natural killer T cells (NKT) were only depleted in year two and did not recover.

**Interpretation:**

Peripheral immune cell profiling revealed more differentiated insights into the immunological effects of CLAD. While some immune cell subsets expanded, we also observed additive depleting effects after the second treatment course. Further studies are required to elucidate whether these changes are paramount for the consistent and prolonged disease‐modifying effect of CLAD.

## Introduction

Multiple sclerosis (MS) is a chronic inflammatory demyelinating disorder of the central nervous system (CNS) with presumed autoimmune etiology. The current understanding of the pathogenesis includes the peripheral activation of myelin‐reactive effector CD4 T helper (TH) 1 cells, memory B cells and TH17 cells.^1^, ^2^, ^3^ Furthermore, there is emerging evidence for a key role of TH17.1 cells, which share inflammatory features of TH17 and TH1 cells.^4^, ^5^

Cladribine (CLAD, MAVENCLAD^®^) is an oral drug approved for treatment of active relapsing‐remitting MS.^6^ This synthetic deoxyadenosine analogue is a prodrug, which selectively depletes immune cells by apoptosis through the caspase system. The cumulative dosage of CLAD tablets in Europe is 3.5 mg/kg divided into four cycles each comprising of 4 or five days depending on body weight over a period of two years.^7^ The mean terminal half‐life with normal renal function is 5.6 h‐7.6 h.^8^ Thus, CLAD is categorized as a pulsed immune reconstitution therapy (IRT), which is defined by short intermittent treatment periods aimed to induce an immune reset and a treatment‐free period due to durable efficacy thereafter.^9^ The flow cytometric analysis of immune cells in peripheral blood of MS patients treated with CLAD revealed a rapid reduction of CD16^+^/CD56^+^ cells (nadir at week 5), a marked reduction in CD19^+^ B cells (nadir at week 13) and a less‐pronounced effect on CD4^+^ (week 13 nadir) and CD8^+^ T cells (nadir at week 24), respectively.^10^ Of note, there are distinct recovery kinetics. B cells return to threshold values by week 84 and CD4^+^ T cells by week 96.^11^ Changes in the proportions of regulatory T cells as well prolonged depletion of central memory CD4 + T cells might contribute to the clinical efficacy on one hand.^10^ On the other hand, it has been hypothesized that the drug‐response relationship with CLAD is more consistent with the B‐depleting effects and related to the depletion of memory B cells. In contrast, there is no or little effect on neutrophils and monocytes.^10^, ^12^

Characterization of immune cell alterations occurring during the disease course and in response to treatment may support a better understanding of MS pathogenesis and the mechanism of action (MoA) of disease‐modifying therapies (DMT). From a therapeutic viewpoint, DMTs may be more effective and associated with lesser extent of side effects if they can specifically correct these detrimental immune processes. Moreover, a sparing of immune cell subsets critical for host defense, immunosurveillance and which foster regenerative processes would be most appreciated. The previous investigations evaluated the impact of CLAD on major immune populations which encompassed only a limited observational period. Further subcategories of T and B cells as well as regulatory lymphocytes have not been studied so far. Here, we aimed to expand the knowledge about depletion and recovery rates of various, closely defined lymphocyte phenotypes following two cycles of CLAD tablets. In this regard, we studied immune cells isolated from the peripheral blood of patients with MS (pwMS) before treatment and every three months thereafter over the course of 2 years by flow cytometric analysis.

## Methods

### Study cohort

CLAD is taken in two treatment courses 1 year apart and each treatment cycle consists of 4 or 5 consecutive days. The recommended cumulative dosage of MAVENCLAD is 3.5 mg/kg body weight administered orally and divided into 2 yearly treatment courses (1.75 mg/kg per treatment course). The use of CLAD was based on the guidance provided by the European Medical Agency.^7^

We collected blood samples by venipuncture from 18 pwMS treated with CLAD at two European MS centers (Christian Doppler Medical Center Salzburg, Austria and Carl Gustav Carus University Hospital, Technische Universität Dresden, Germany). The blood sampling was approved by the local Ethics Committees and all patients provided written consent for the usage of their biological samples for research purposes. We collected demographic data, medical history and details of the previous MS course, and assessed the Expanded Disability Status Score (EDSS). Neurological examination was performed and blood samples were taken at baseline (BL) and every 12 weeks (±4 weeks) for up to 24 months.

### Routine blood testing

Whole blood samples were collected in ethylene diamine tetra acetic acid (EDTA) tubes and testing was performed at the Institutes of Laboratory Medicine of University Hospital in Dresden, Germany and Christian Doppler Medical Center Salzburg, Austria, respectively. The institutes comply with the standards required by DIN‐EN‐ISO‐15189:2014 for medical laboratories. Complete blood cell counts were used to define absolute numbers of lymphocyte subgroups including T lymphocytes, TH cells, cytotoxic T cells, B cells, natural killer cells as well as of innate immune cells (monocytes). Peripheral blood mononuclear cells (PBMCs) were calculated as sum of absolute numbers of lymphocytes and monocytes.

### Immune cell phenotyping by fluorescence‐activated cell scanning (FACS)

PBMCs were isolated and processed according to standard operating procedures (SOPs) as described before.^13^ In short, PBMCs were isolated from heparinized or citrated blood and cryo‐preserved at −130°C. After thawing, cells were prepared at a concentration of 2x10^6^ cells/mL in human AB serum.

For surface staining (min. 400 µL), cells were incubated with the viability marker Zombie Green–Alexa488 (Biolegend, San Diego, CA, USA) for 20 min and washed with FACS buffer (phosphate buffered saline, 0.2% fetal calf serum, 0.02% sodium azide). Subsequently, subpopulations were identified and quantified by surface staining with fluorescence labeled antibodies (BD‐Biosciences, Franklin Lakes, NJ, USA: CD3‐APC‐H7, CD4‐PE‐C7, CD8PerCPCy5, CD10‐BV786, CD19‐BV510, CD21‐APC, CD25‐BV421, CD27‐BV605, CD38‐A700, CD45RA‐APC, CD45RO‐BV605, CD127‐BV510, CD197‐PE‐CF594, CXCR3‐PE‐Cy5, IgM‐PeCy5, CD138‐BV421; Biolegend, San Diego, CA, USA: CD5‐PECy7, CD14‐BV711, CD16‐A700, CD20‐BV711, CD24‐PE, CD56‐BV785, CD196‐PE, IgD‐PerCP‐Cy5.5). Corresponding isotype antibodies were used as negative controls.

For intracellular staining, freshly thawed PBMCs were suspended in human AB serum overnight and subsequently stimulated with 10ng/mL phorbol myristate acetate (PMA, Sigma‐Aldrich, St. Louis, MO, USA) and 1µg/mL ionomycin (Sigma‐Aldrich) and supplemented with 0.2µM monensin (Biomol, Hamburg, Germany) for 6 h at 37°C. After harvesting, cells were incubated with viability dye staining (Zombie Green–Alexa488) for 20 min and after corresponding washing surface staining was performed (BD‐Biosciences: CD3‐APC‐H7, CD4‐BV510, CD196‐BV605, CD197‐A700, CXCR3‐PE‐Cy5; Biolegend: CD20‐BV711). Cells were then fixed with paraformaldehyde (PFA) and permeabilized with saponin before being incubated with intracellular fluorescence labeled antibodies (BD‐Biosciences: IL‐10‐PE‐CF594, TNF‐α‐BV421; Biolegend: IL‐17A‐PE, GM‐CSF‐APC, IFN‐γ‐BV786; Thermofisher, Waltham, MA, USA: IL‐22‐PE‐Cy7) for 30 min. All samples were rinsed with FACS buffer and measured on LSR‐Fortessa (BD Biosciences). FACS‐Diva Software (BD‐Bioscience) was used for data analysis. An overview on the definition of the immune cell subsets is shown in Table S1.

### Statistical analysis

For statistical analysis, we used IBM SPSS Statistics 25 Software for Windows (Version 25.0; IBM Corporation, Armonk, NY, USA). After testing for normal distribution by Shapiro‐Wilk test, data were analyzed by Generalized Linear Mixed Models (GLMM) with Bonferroni correction. If data exhibited a right‐skewed distribution pattern gamma distribution was used. The percentual changes of immune cell subsets in the course of treatment were calculated from absolute numbers in comparison to baseline. P‐values were considered significant as follows: *P ≤ 0.05, **P ≤ 0.01, ***P ≤ 0.001 and ****P ≤ 0.0001. Graphs were created with GraphPad PRISM 8 (Graphpad Software, San Diego, CA, USA).

## Results

### Demographics

We recruited 18 patients, of whom 15 (83%) were women. The mean age was 37 years and 14 (78%) received another immunomodulatory treatment prior to CLAD. The washout period followed the general guidelines and the lymphocyte counts were normal before starting therapy with CLAD. The number of patients in whom PBMCs were collected at each timepoint was as follows: baseline throughout month 9: *n* = 18, month 12: *n* = 16; month 15: *n* = 10; month 18: *n* = 9; months 21 and 24: *n* = 4. Further details of demographics are shown in Table 1 and the scheme for blood sampling is presented in Figure S1.

**Table 1:** Baseline cohort characteristics.

	n = 18
Sex F/M	15/3
Age, mean years ± SD (range)	37.4 ± 11.7 (20–57)
EDSS BL, mean ± SD	2.25 ± 1.7
EDSS EOS, mean ± SD	2.08 ± 2.0
MS duration, mean years (range)	8.8 (0‐25)
Immunmodulatory treatment before CLAD, n (%)	14 (78)
Glatirameracetate	3 (17)
Interferon‐β	2 (11)
Teriflunomid	1 (6)
Dimethylfumarate	2 (11)
Daclizumab	2 (11)
Fingolimode	3 (17)
IVIG	1 (6)

F, female; M, male; SD, standard deviation; BL, baseline; EOS, end of study; CLAD, cladribine; IVIG, intravenous immunoglobulin.

### Main immune subsets

**Figure 1:**
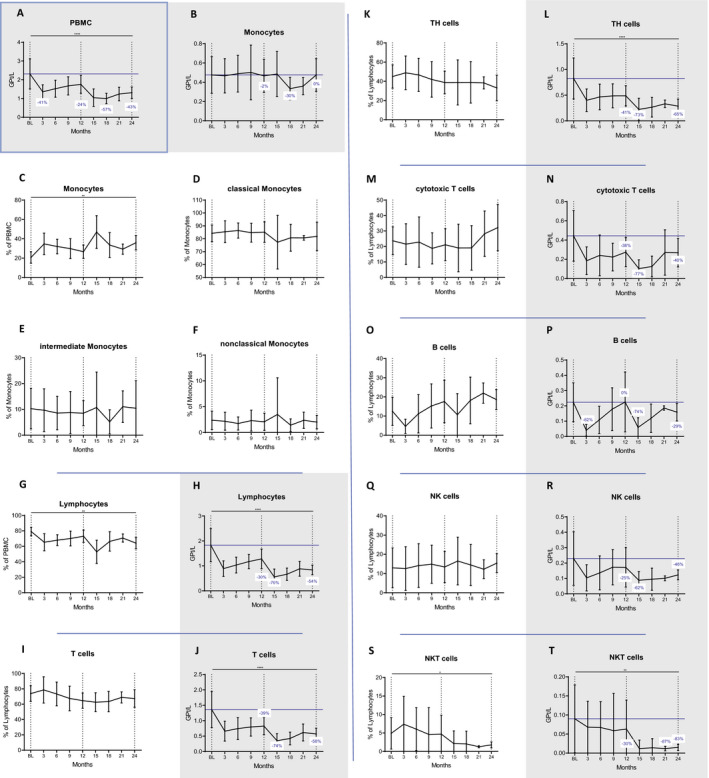
Longitudinal monitoring of repopulation kinetics of immune cell subsets after treatment with CLAD. CLAD intake was prior to BL and after 12 months (dotted lines, y‐axis). (A) Displayed are temporal dynamics of peripheral blood mononuclear cells (PBMCs) over 24 months. We studied absolute (GPt/L) and relative (%) changes. In this regard, monocytes were spared (B to F), while lymphocytes are significantly reduced (G and H). B cells are strongly reduced after each CLAD treatment but followed by recovery towards the end of each year (O and P). In contrast, the reduction of T‐cell subsets (I and J, K to N) and NK/NKT cells (Q to T) is more persistent and for some subsets more extensive in the second year. The horizontal line shows BL values; blue numbers show changes in cell counts compared to BL. Data are shown as mean values +/‐SD. *P ≤ 0.05, **P ≤ 0.01, ***P ≤ 0.001 and ****P ≤ 0.0001.

We first investigated the CLAD‐induced changes of the main immune cell subsets. As compared to baseline, PBMCs were significantly reduced at any investigated timepoint and were pronounced in the second year (at month 24 *P* = 0.00000009, Fig. 1A). While the proportion of monocytes significantly increased (at month 24 *P* = 0.0011, Fig. 1B and C), we did not find changes in the three major subsets. These included classical (CD14^++^CD16^−^), intermediate (CD14^+^CD16^+^) and nonclassical (CD14^low^CD16^++^) monocytes, (Fig. 1D to F).

Lymphocyte proportions and counts significantly decreased after CLAD administration (at month 24 *P*=0.0013 and *P*=0.000003, respectively, Fig. 1G and H). The nadir was reached at month 15 (−70%) and lymphocyte counts were in general lower in year 2 (−30% and −54% at month 12 and 24, respectively). This observation was especially true for CD3^+^ T cells, CD3^+^CD4^+^ TH cells and CD3^+^CD8^+^ cytotoxic T cells (cT, Fig. 1I to N). The former two cell subsets were affected to a similar extent at the end of year one (−40%), reached a nadir at month 15 (−73% with *P* = 0.000000038 and −74% with *P* = 0.00001, respectively) and slowly recovered to −58% and −65% from BL, respectively at month 24. By that time, decreases in cell counts were still highly significant (*P* = 0.000049 and *P* = 0.00006 for CD3^+^ and CD3^+^CD4^+^, respectively). CD8 cells were depleted to a similar extent at their nadir at month 15 (−77%, *P* = 0.000027) but recovered faster (−40% at month 24, *P* = 0.142) in year two, when they exhibited a tendency of expansion among the lymphocyte pool (Fig. 1M).

The nadir of CD19^+^ B cells was at three months after each treatment cycle (*P* = 0.00000045 and *P* = 0.00009, respectively; Fig. 1O and P). Contrasting the findings of T cells, the depletion was more effective in year one (−82% vs. −74%). The recovery kinetics of CD19^+^ B cells differed as well. B‐cell counts reached pretreatment levels at the end of year one and were reduced by 29% compared to BL at the end of year two. In fact, their proportions expanded among lymphocytes after the early depletion phase, exceeding BL values from month 6 onwards (*P* = 0.025 and *P* = 0.320 for 21 and 24 months, respectively).

Natural killer cells (NK, CD56^+^CD3^−^) were significantly depleted (*P* = 0.033 and *P* = 0.016 at month 3 and 15, respectively) but recovered to reach −25% and −46% of BL at the end of each cycle. The frequency in the lymphocyte pool remained unchanged (Fig. 1Q and R) and the nadir was reached by month 15 (−62%). In contrast, CD56^+^CD3^+^ natural killer T cells (NKT) were selectively depleted in year two, when their reductions were most pronounced among the major lymphocyte subsets. By month 24, their proportions among lymphocytes and their counts (−83% compared to BL) were significantly decreased (p=.032 and p=.0052, respectively; Fig. 1S and T).

The proportional changes of the major immune cell subsets within PBMCs over 24 months are shown in Figure S2A.

### T‐cell subsets

**Figure 2:**
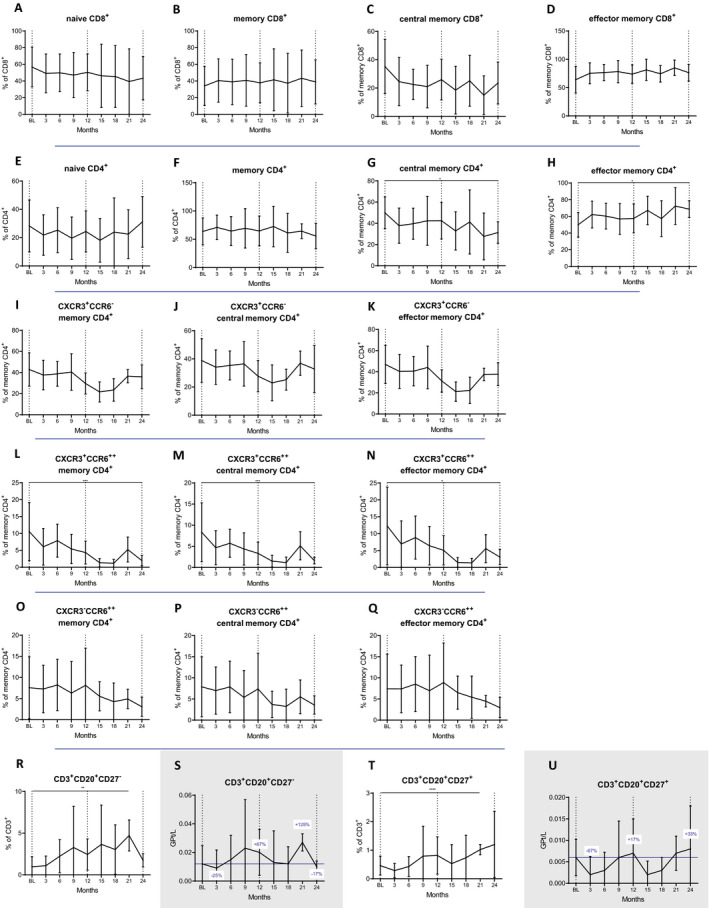
Impact of CLAD on different T‐cell subsets over 24 months: cT (CD8, A to D), TH (CD4, E to H) and CD3+  CD20+ T cells (R to U). We found a persistent depletion of central memory TH cells (CD45RO + CCR7+, G) and an expansion of the effector memory TH cells (CD45RO + CCR7‐, H). All subsets of TH17.1 cells (CD3 + CD4+CXCR3 + CD196high) were significantly decreased and changes were more extensive after the second year (memory, central memory and effectors memory CD4, (O to Q). On the other hand, CD20 + T‐cell subsets significantly expanded over the observation period (R‐U). Vertical dotted lines indicate baseline (BL) and the end of each treatment year, blue horizontal line shows BL values; blue numbers show changes in cell counts compared to BL. Data are shown as mean values +/‐SD. *P ≤ 0.05, **P ≤ 0.01, ***P ≤ 0.001 and ****P ≤ 0.0001.

We next investigated the kinetics of T‐cell phenotypes according to different maturation markers. Within the group of cytotoxic T cells, the proportions of naive (CD8^+^CD45RA^+^) and memory (CD8^+^CD45RO^+^) cells were not altered by CLAD (Fig. 2A and B). This was also not the case for central memory cells (cm, CD8^+^CD45RO^+^CCR7^+^) and their effector memory counterparts (em, CD8^+^CD45RO^+^CCR7^−^, Fig. 2C and D). Also among TH cells, the proportions of naive and memory subsets were not altered (Fig. 2E and F). Importantly, the proportions of cmTH cells (CD4^+^CD45RO^+^CCR7^+^) were significantly reduced by CLAD (at month 24 *P* = 0.025; Fig. 2G), while effector memory proportions increased (at month 24 *P* = 0.024; Fig. 2H).

Next, we determined the impact of CLAD on different TH subsets defined by surface markers which enable the distinction of TH1, TH17.1 and TH17 cells. Regarding their proportions among TH cells (Fig. 2I to Q), CLAD exerts a selective effect on central memory TH17.1 cells (CD4^+^CD45RO^+^CCR7^+^CXCR3^+^CD196^high^), which were strongly reduced (*P* = 0.000796 at month 24, Fig. 2M). There were no statistical differences for TH1 (CD4^+^CD45RO^+^CXCR3^+^CD196^−^; Fig. 2I to K) and TH17 (CD4^+^CD45RO^+^CXCR3^−^CD196^high^; Fig. 2O to Q) phenotypes over the observation period. In absolute terms, however, CLAD depleted TH1 (−78%, *P* = 0.0000162), TH17 (−93%, *P* = 0.156) and TH17.1 immune cell subsets (−96%, *P* = 0.035) at the end of year two (data not shown).

CD20^+^ T‐cell subsets (Fig. 2R to U) were expanded at month 21 (CD27^−^: *P* = 0.002; CD27^+^: *P* = 0.000077).

### B‐cell subsets

**Figure 3:**
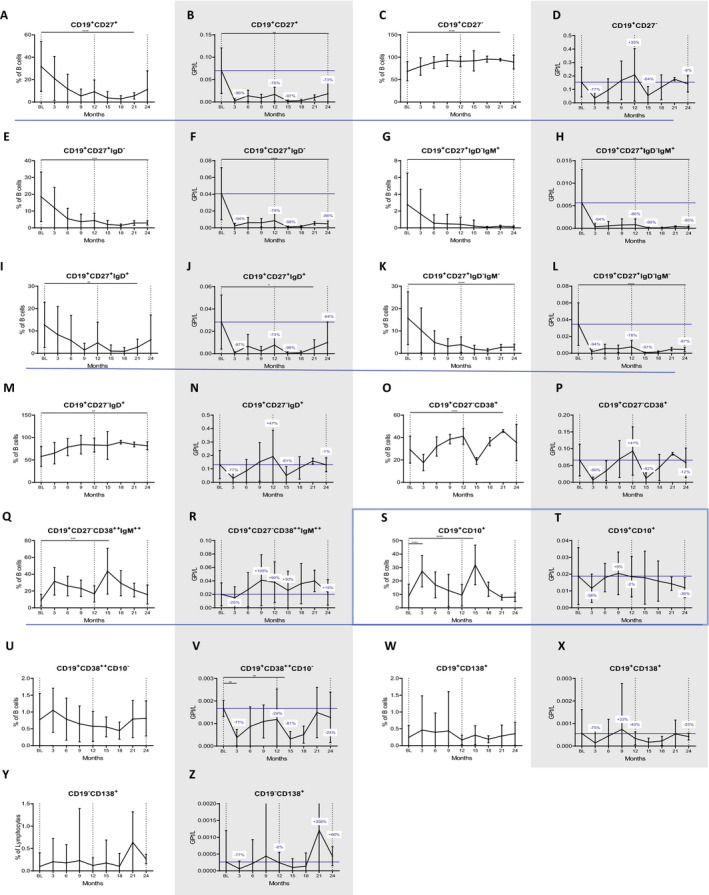
Impact of CLAD on different B‐cell subsets over 24 months. There was a significant decrease of memory B cells which remained below BL values at the end of the observation period (A and B). Reductions were pronounced in IgD‐ subsets (E to H, K and L), while unswitched memory B cells started to expand towards the end of year two (I and J). On the contrary, proportions of naive B cells expanded (C, M, O and Q) and after an initial depletion, CD27‐ B‐cell counts hyper‐repopulated (D, N, P, R). There was a transient relative increase of immature B cells (S) and even lower absolute number at month 24 (−38%, T). Proportions of antibody‐producing B cells were not significantly altered (U, W and Y), and after an initial depletion, plasmablasts exhibited fast recovery rates (V and X) and plasma cells even exceeded BL values at the end of each cycle (Z). Vertical dotted lines indicate baseline (BL) and the end of each treatment year, the blue horizontal line shows BL values; blue numbers show changes in cell counts compared to BL. Data are shown as mean values +/‐SD. *P ≤ 0.05, **P ≤ 0.01, ***P ≤ 0.001 and ****P ≤ 0.0001.

We then studied the kinetics of the different B subsets over 24 months. We found that CLAD strongly reduced memory B cell (CD19^+^CD27^+^) counts (−76% with *P* = 0.000068 and −73% with *P* = 0.0055 at the end of year one and two respectively; Fig. 3B) and proportions (*P* = 0.000028 at month 21; Fig. 3A), resulting in a relative expansion of naive B cells (CD19^+^CD27^−^, *P* = 0.000025 and *P* = 0.05 by months 21 and 24, respectively; Fig. 3C and D). Regarding the early impact of CLAD (months 3 and 15) on antigen experienced B cells (CD27^+^), we found that all phenotypes were depleted by at least 94% (Fig. 3E to L). When analyzing the repopulation of the various memory B‐cell subsets towards the end of each treatment year, IgM‐only expressing memory B cells (CD19^+^CD27^+^IgD^−^IgM^+^, −86% *P* = 0.016, −95% *P* = 0.016 for months 12 and 24, respectively; Fig. 3H), and CD19^+^CD27^+^IgD^−^IgM^−^ class‐switched memory B cells (−78% with *P* = 0.00004 and‐ 87% with *P* = 0.000019, for months 12 and 24, respectively; Fig. 3L) remained significantly depleted. On the other hand, unswitched memory B cells (CD19^+^CD27^+^IgD^+^) recovered faster and CLAD‐induced reductions were not significant at month 24 anymore (*P* = 0.093; Fig. 3J). We continued with the evaluation of naive B‐cell subsets. CD19^+^CD27^−^ phenotypes were initially reduced after CLAD administration but unlike memory B cells they completely recovered within 9 months of each cycle, resulting in a mild hyper‐repopulation at the end of year one (+35% form BL, Fig. 3D). The recovery rates were even more pronounced looking at certain naive B‐cell subsets (Fig. 3M to R), which all significantly expanded in relative terms (CD19^+^CD27^−^IgD^+^: *P* = 0.003 at month 24; CD19^+^CD27^−^CD38^++^IgM^++^: *P* = 0.0008 at month 15; CD19^+^CD27^−^CD38^+^ *P* = 0.0000027 at month 21) and counts temporarily even reached values twice as high compared to BL (statistically not significant). The highest numbers were reached in the second half of each treatment year. We then investigated the impact on immature B cells (CD19^+^CD10^+^), which expanded from 9% at BL to 27% within the first three months (*P* = 0.0000053), followed by a decline to reach BL values at the end of year one – before repeating the same kinetics in year two (Fig. 3S and T).

Lastly, CLAD did not induce significant changes in the proportions of antibody‐producing B cells (Fig. 3U to Z), namely plasmablasts (CD19^+^CD138^+^ or CD19^+^CD38^++^CD10^−^) and plasma cells (CD3^−^CD19^−^CD138^+^). In absolute terms, CD19^+^CD38^++^CD10^−^ were significantly depleted following each administration (*P* = 0.006 and *P* = 0.004 at month 3 and 15) and recovered to values approximate to BL, while absolute changes in CD19^+^CD138^+^ and CD19^−^CD138^+^ were statistically not significant. Both phenotypes were initially depleted by CLAD, followed by a complete recovery, and the latter even showed a trend towards hyper‐repopulation in year two.

The proportional changes of B‐cell phenotypes over 24 months is shown in Figure S2B.

### Regulatory immune cell subsets

**Figure 4:**
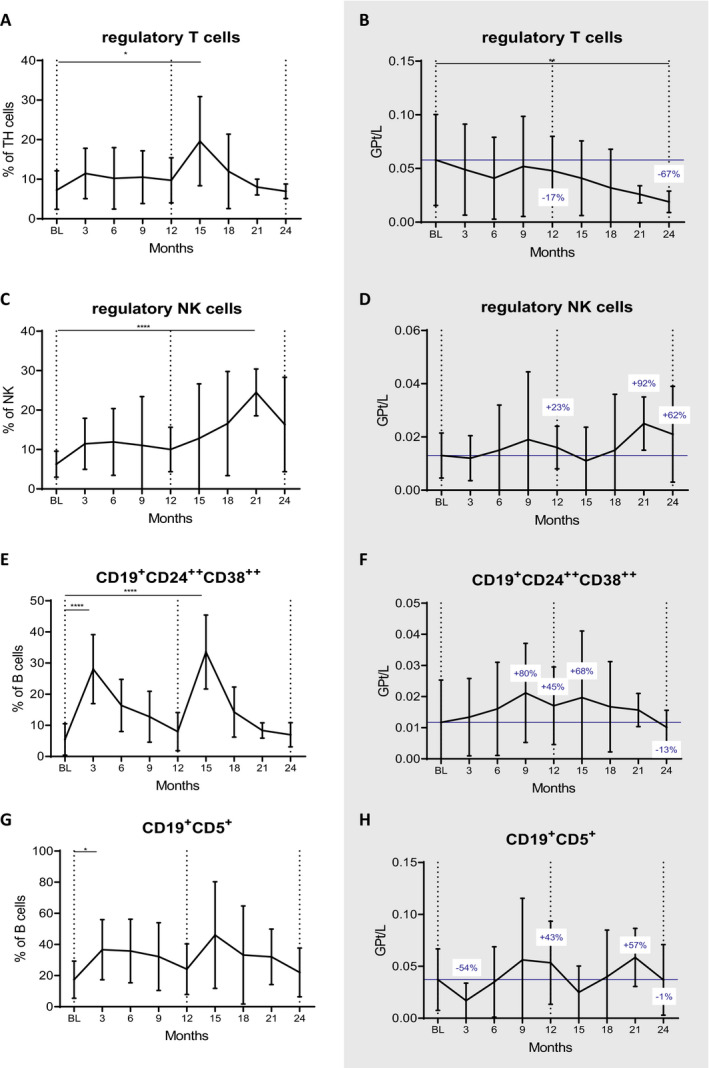
Impact of CLAD on different regulatory immune cell subsets over 24 months. The proportion of Tregs were relatively unaffected in the first year (A), while absolute numbers significantly decreased towards the end of the observational period (B). Regulatory NK cells increased in relative (C) and absolute (D) terms. Regulatory B cells expanded shortly after each treatment cycle (E and G) and kinetics regarding cell counts varied according to the gating strategy. Both approaches showed a trend towards hyper‐repopulation (F and H). Vertical dotted lines indicate baseline (BL) and the end of each treatment year, blue horizontal line shows BL values; blue numbers show changes in cell counts compared to BL. Data are shown as mean values +/‐SD. *P ≤ 0.05, **P ≤ 0.01, ***P ≤ 0.001 and ****P ≤ 0.0001.

We moved on to study the major regulatory immune cell subsets. The proportions of regulatory T cells (Tregs, CD4^+^CD25^++^CD127^−^) expanded at month 15 (*P* = 0.011), followed by a steady decrease approximating pretreatment values by month 24 (Fig. 4A). By this time, the Treg counts were reduced by 67% compared to BL (*P* = 0.006, Fig. 4B).

Expansions were more pronounced in the regulatory NK cell (CD56^++^CD16^low^) subset. We found that the proportion of this subset significantly increased already at three months and peaked at 21 months (p = 0.022 and *P* = 0.00000039, Fig. 4C). In absolute terms, they showed a tendency of expansion, which was most pronounced at month 21 (+92%, statistically not significant; Fig. 4D).

Regulatory B cells (Bregs, CD19^+^CD24^++^CD38^++^) proportions increased shortly after each administration (*P* = 0.00000000004 and *P* = 0.000000001 at three and 15 months respectively; Fig. 4E). Also CD19^+^CD5^+^ Bregs significantly expanded after CLAD start (*P* = 0.004 at month 3; Fig. 4G). Absolute numbers exceeded BL values by 43% and 45% at month 12 (statistically not significant) and were just below pretreatment levels at the end of the following year (Figure F and H).

### Intracellular cytokine expression of TH1, TH17 and TH22 subsets

**Figure 5:**
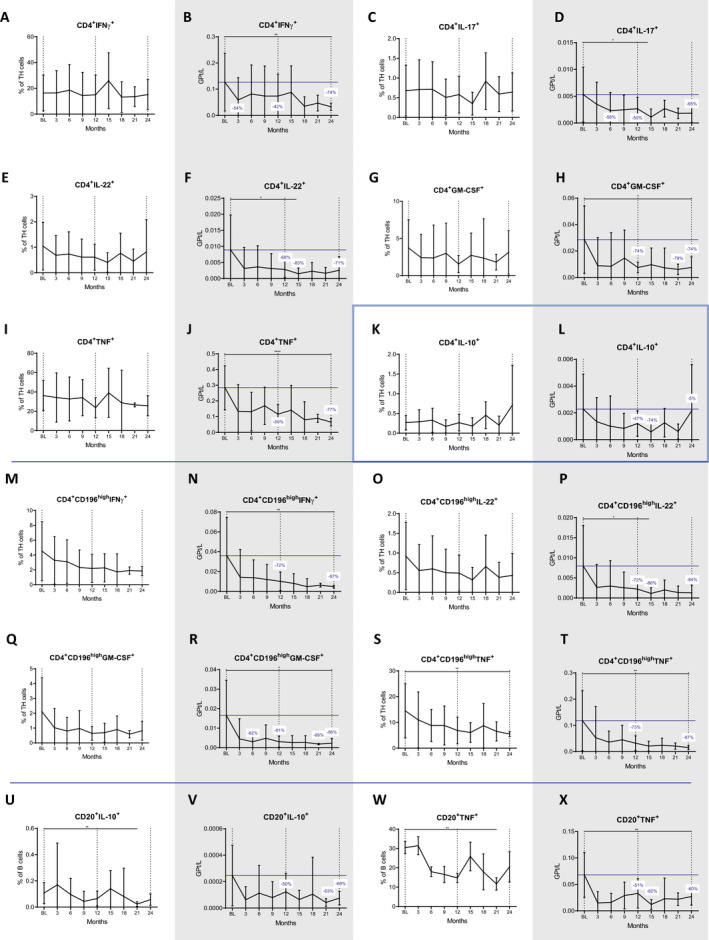
Impact of CLAD on intracelular cytokine production in TH1 (A to L) and TH17 (M to T) subsets and B cells (U to X). Among TH17‐like cells (CD196high), only proportions of those expressing TNF‐α (S) significantly decreased, while reductions of IFN‐γ (M), IL‐22 (O) and GM‐CSF (Q) producers did not reach statistical significance. In terms of absolute numbers all subsets were strongly depleted, and decreases were more marked in year two (B, D, F, H, J, N, P, R and T). B cells expressing TNF‐α and IL‐10 were significantly lowered at month 21 (U and W) and also their absolute counts were reduced (V and X). Vertical dotted lines indicate baseline (BL) and the end of each treatment year, the blue horizontal line shows BL values; blue numbers show changes in cell counts compared to BL. Data are shown as mean values +/‐SD. *P ≤ 0.05, **P ≤ 0.01, ***P ≤ 0.001 and ****P ≤ 0.0001.

We measured the cytokine expression of stimulated lymphocytes by intracellular staining. For definition means, we gated TH1 cells as CD3^+^CD4^+^IFN‐γ^+^, TH17 cells as CD3^+^CD4^+^IL‐17^+^ and TH22 as CD3^+^CD4^+^IL‐22^+^ (Fig. 5A to F); additionally, we explored the kinetics of CD3^+^CD4^+^ cells producing GM‐CSF and TNF‐α (Fig. 5G to J). Nonclassical TH17 cells were defined as CD3^+^CD4^+^CD196^high^ cells and evaluated for expression of GM‐CSF, IFN‐γ, IL‐22, TNF‐α (Fig. 5M to T).

Many of the TH phenotypes decreased after the first year and had a more pronounced nadir after the second year of treatment. These included TH1 cells which were reduced by 42% and 74% (*P* = 0.005 at month 24; Fig. 5B), TH17 cells which were lowered by 50% and 65% (*P* = 0.135 at 24 months; Fig. 5D) and TH22 cells which dropped by 68% and 71% (*P* = 0.174 at month 24; Fig. 5F), respectively. Reductions of the latter two were significant at month 15 (*P* = 0.014 and *P* = 0.047, respectively).

Depletion rates were most marked for the CD196^high^ TH17‐like cells. Herein, IFN‐γ expressing cells were reduced by 72% and 87% (*P* = 0.007 at month 24; Fig. 5N), GM‐CSF producing cells by 81% and 86% (*P* = 0.011 at month 24; Fig. 5R) and TNF‐α producing cells by 73% and 87% (*P* = 0.002 at the end of year two; Fig. 5T). CLAD had no significant effect on CD4^+^ cells producing IL‐10, an anti‐inflammatory phenotype (Fig. 5K and L). There was a lowering of nonclassical TH17 cells producing TNF‐α (*P* = 0.005 at 24 months, Figure 5S).

The study of the cytokine expression of CD20^+^ B cells (Fig. 5U to X) revealed that the proportions of TNF‐α expressing B cells were significantly reduced (*P* = 0.007 at the end of year two; Fig. 5W). Interestingly, also antiinflammatory, IL‐10 expressing B cells were numerically decreased (*P* = 0.028 at 24 months). IL‐17 producing CD20^+^T cells were not significantly altered during the entire observation period (data not shown).

## Discussion

**Figure 6:**
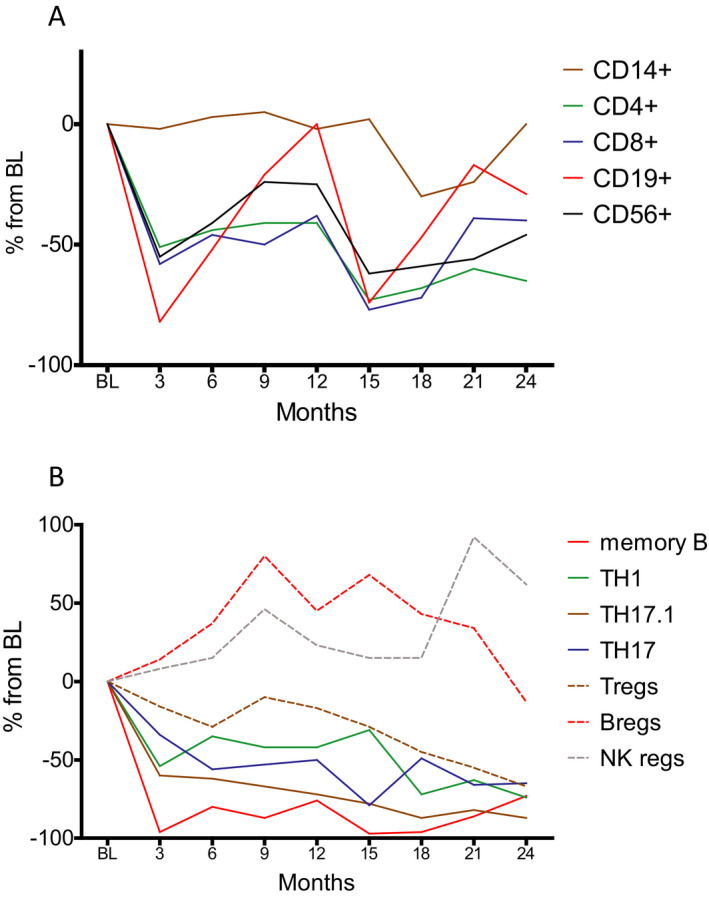
Dynamics of PBMC subsets after treatment with two cycles of CLAD. We distinguished (A) the major culprits of MS (solid lines) and (B) potentially beneficial cell lineages (dashed lines). Percentages are referred to as changes in absolute counts as compared to baseline (BL) values. Vertical dotted lines indicate BL and the end of each treatment year.

In this study, we report several novel findings regarding the immunological consequences in the peripheral compartment after intake of oral CLAD in pwMS. These are of major interest since IRTs including CLAD, alemtuzumab (ALEM), anti‐CD20 antibodies and autologous hematopoietic stem cell transplantation (AHSCT) deplete components of the immunological repertoire with the aim of allowing the immune system to renew itself in the absence of ongoing direct drug effects.‐ In this regard, we corroborate the different depletion kinetics on peripheral immune cell subsets of the adaptive immune system (Fig. 6A).^10^, ^11^ This selectivity can be explained by virtue of the drug’s biology and the distribution of deoxycytidine kinase (DCK), which activates CLAD by phosphorylation and is highly expressed in lymphocytes.^14^ Moreover, we show that some of the immunological changes are more pronounced or exclusively in the second year, which can be seen as a consequence of the second annual treatment cycle.

In detail, CLAD leads to profound changes within the B‐cell population, where it exhibits dramatic reductions of class‐switched memory B cells with a concomitant increase in maturing B cells. The impact on memory B cells was similar to the depletion rates found after treatment with injectable CLAD^15^ and has been implicated in the long‐lasting efficacy of IRTs.^1^, ^9^, ^16^, ^17^ We also confirm a selectivity of CLAD towards central memory TH cells. Importantly, a majority of autoreactive cells belong to this very phenotype.^18^ Moreover, central memory TH cells are the main targets of alemtuzumab and fingolimod, which are two other efficacious treatment options for active MS.^19^, ^20^, ^21^ Regarding TH subsets, we could not identify significant changes in the proportions of TH1, TH22 and various TH17 subsets as defined by cytokine expression. This is somewhat surprising, as CLAD showed inhibitory action on T‐cell cytokine expression independent of immune cell depletion in an vitro model.^22^ However, it is of paramount importance to interpret relative changes in the context of depletion kinetics of the respective parent lymphocyte population. While B cells approximate pretreatment values towards the end of each treatment year, TH cells remain clearly below baseline levels throughout the 24 month investigational period. Therefore, the apparent selective depletion of memory B cells is biased by the (hyper‐) repopulation of maturing B cells. It hence appears to be more appropriate to compare absolute changes of immune subsets.

We demonstrate a nearly complete depletion of memory B cells at three months from CLAD administration followed by a slow repopulation. In addition, there is a stepwise but strong reduction of TH17 and even more pronounced of nonclassical TH17 cells. Consequently, our long‐term data reveal that memory B cells and nonclassical TH17 subsets are modulated by CLAD and reduced to a similar extent at 24 months from treatment start (Fig. 6B). Among all examined immune cell subsets in this study, these were reduced the most in the long‐term investigation.

Importantly, both immune cell subsets are among the main orchestrators of the immune pathogenesis of the disease.^1^, ^2^, ^4^, ^23^, ^24^ Moreover, the surface staining approach shows a paramount impact on central memory TH17.1 cells, which are as well closely linked to MS pathogenesis.^4^, ^25^ Of note, reductions of TH1 and TH22 cells as well as TH17 subsets were most pronounced in year two. Our data suggest that the efficacy of CLAD may be potentiated by the second cycle. In contrast to TH recoveries, B‐cell kinetics including memory B cells did not substantially vary between year one and two.

With regard to CLAD effects on the B‐cell lineage, we show that their repletion kinetics are reminiscent of B‐cell recovery following ALEM, although to a lesser extent. For this CD52‐depleting antibody approved for active MS, an early increase in immature B cells is followed by hyper‐repopulation of naive B cells.^26^ This is of particular interest since the ALEM‐associated B‐cell repopulation kinetics are suspected to induce secondary autoimmunity, which however is not seen with CLAD. Our data therefore either relativize the blame of immature B cells as possible substrates, or support the theory, that autoreactive clones only become pathogenic in the presence of a marked, simultaneous lymphopenia.^27^ While ALEM induces a nearly complete depletion of peripheral lymphocytes within one week of administration,^28^ the lymphocyte reductions following CLAD are more protracted and the nadir is reached beyond the first month.^10^

The slower depletion rates combined with our findings that regulatory B, T and NK immune cell subsets undergo expansion are of major interest. This observation might relate to an attempt of the repleted immune system to restore a homeostatic immunological environment, and eventually suppress autoreactive lymphocytes. The preservation of antiinflammatory lymphocytes might additionally contribute to the therapeutic value in pwMS.

The analysis of immune cell subsets important for infection control revealed that cytotoxic T cells and NK cells are only modestly affected, monocytes are spared, and antibody‐producing B cells recover towards the end of each treatment year. These kinetics suggest that patients receiving CLAD may be more prone to viral infections within the first 9 month of administration, which should be considered for clinical purposes. In this regard, prophylactic acyclovir therapy is recommended in patients experiencing a grade IV lymphopenia following CLAD.^29^ As antibodies contribute to maintain protection from pathogens, inhibition of (intrathecal) antibody production is of special interest in the pathomechanisms involved in MS, where oligoclonal bands (OCBs) represent a hallmark. In fact, injectable CLAD was found to eliminate OCBs in 55% of pwMS, which in turn correlated with better clinical outcome.^30^ This finding not only provided evidence that CLAD, as a small molecule, reaches the CNS,^31^, ^32^ but also that CNS penetration is associated with functional consequences. In contrast to circulating plasma cells, antibody‐producing cells within the CNS might recover in a slower fashion and subsequently contribute to the long‐term efficacy of CLAD.

The main limitation of this study is the decline of patient numbers over the observation period and the lack of a control group. Moreover, there might be further immunological consequences beyond 24 months from initiation of CLAD therapy. Thus, our findings need to be confirmed in a larger cohort and further studies should extend the observation period. Cryopreservation, a standard procedure for immune cell storage, allows measurements of various (blood) samples collected over time under equal circumstances. However, it should be taken into considerations that some immune populations may be more susceptible to cell death after freezing and thawing.^33^

To conclude, our results suggest a selectivity of CLAD towards proinflammatory immune cell subsets, resulting in long‐term depletion of memory B cell and TH17 cell populations, while cells involved in immune reactions against pathogens are less affected. We also expand the knowledge about the mode of action by reporting a proportional increase of different regulatory cells and more extensive depletion of certain immune cells subsets in the second year, which indicates a potential additive effect of the two annual treatment courses.

## Conflicts of interest

TM, KS, MS, JF, and EHB report no disclosures. KA received honoraria for presentations or participation on advisory boards from Biogen Idec, Merck, Sanofi, and Roche. TZ received personal compensation from Almirall, Biogen Idec, Bayer, Celgene, Novartis, Roche, Sanofi, and Teva for consulting services. His institution received financial support for research activities from BAT, Biogen, Novartis, Roche, Teva, and Sanofi Aventis. JS received honoraria for consultancy or participation in advisory boards from Alexion, Celgene, Merck, Novartis, Immunic, Sanofi, and Roche.

## Supporting information


**Figure S1.** Schedule for drug intake, neurological examinations and blood sampling: baseline (BL) and every 12 weeks for up to 24 months.

**Figure S2.** (A) Proportional changes of the major immune cell subsets within PBMCs over 24 months, and (B) proportional changes of B cell phenotypes over 24 months.

**Table S1.** Definition of immune cell subsets for FACS analysis.
