# Supplementary figures and images for: Long‐term peripheral immune cell profiling reveals further targets of oral cladribine in MS
Source: Ann Clin Transl Neurol. 2020 Oct 1;7(11):2199–212. doi: 10.1002/acn3.51206 (PMC7664268; doi:10.1002/acn3.51206)

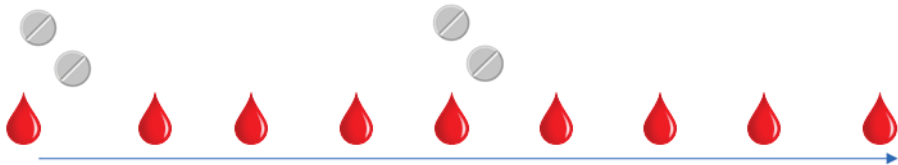

months

0

3

6

9

12

15

18

21

24

n=

18

16

10

9

4

Supplement: Supplementary file 1 — Figure S1. Schedule for drug intake, neurological examinations and blood sampling: baseline (BL) and every 12 weeks for up to 24 months. [file ACN3-7-2199-s001.pdf]

# PBMC

A

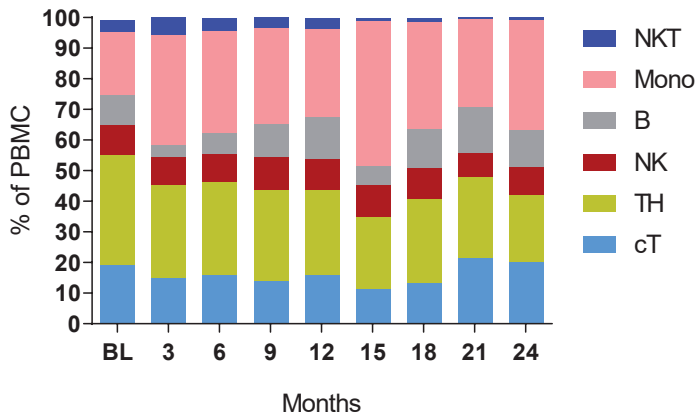

B

# B cells

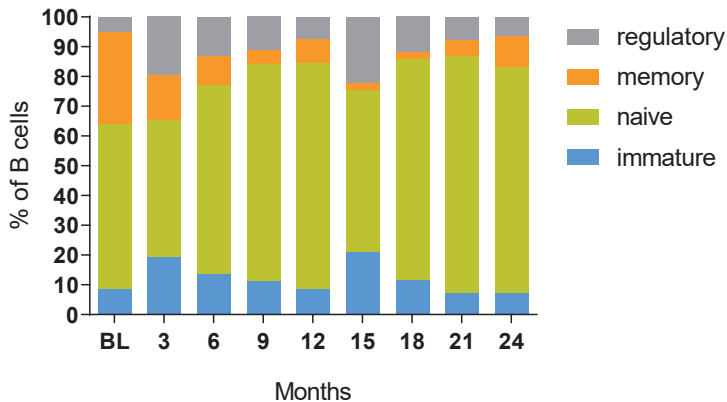

Supplement: Supplementary file 2 — Figure S2. (A) Proportional changes of the major immune cell subsets within PBMCs over 24 months, and (B) proportional changes of B cell phenotypes over 24 months. [file ACN3-7-2199-s002.pdf]
