# Supplementary material for: Long‐term peripheral immune cell profiling reveals further targets of oral cladribine in MS
Source: Ann Clin Transl Neurol. 2020 Oct 1;7(11):2199–212. doi: 10.1002/acn3.51206 (PMC7664268; doi:10.1002/acn3.51206)
Supplement: Supplementary file 3 — Table S1. Definition of immune cell subsets for FACS analysis. [file ACN3-7-2199-s003.docx]

**Supplementary Table 1**: Definition of immune cell subsets for FACS analysis

|  |  |  |
| --- | --- | --- |
| **T cells** | **CD3+CD56-CD14-** |  |
|  | CD3+CD4+ | TH cell |
|  | CD3+CD4+CD45RA+ | Naïve TH cell |
|  | CD3+CD4+CD45RO+ | Memory TH cell |
|  | CD3+CD4+CD45RO+CCR7+ | Central memory TH cell |
|  | CD3+CD4+CD45RO+CCR7- | Effector memory TH cell |
|  | CD3+CD4+CXCR3+CD196- | TH1 cell |
|  | CD3+CD4+CXCR3-CD196high | TH17 cell |
|  | CD3+CD4+CXCR3+CD196high | TH17.1 cell |
|  | CD3+CD4+CXCR3+CD196-CD45RO+ | Memory TH1 cell |
|  | CD3+CD4+CXCR3-CD196highCD45RO+ | Memory TH17 cell |
|  | CD3+CD4+CXCR3+CD196highCD45RO+ | Memory TH17.1 cell |
|  | CD3+CD4+CXCR3+CD196-CD45RO+CCR7+ | Central memory TH1 cell |
|  | CD3+CD4+CXCR3+CD196-CD45RO+CCR7- | Effector memory TH1 cell |
|  | CD3+CD4+CXCR3-CD196highCD45RO+CCR7+ | Central memory TH17 cell |
|  | CD3+CD4+CXCR3-CD196highCD45RO+CCR7- | Effector memory TH17 cell |
|  | CD3+CD4+CXCR3+CD196highCD45RO+CCR7+ | Central memory TH17.1 cell |
|  | CD3+CD4+CXCR3+CD196highCD45RO+CCR7- | Effector memory TH17.1 cell |
|  | CD3+CD4+CD25highCD127- | Regulatory T cell |
|  | CD3+CD4+CD25highCD127-CD45RA+ | Naïve regulatory T cell |
|  | CD3+CD4+CD25highCD127-CD45RO+ | Memory regulatory T cell |
|  | CD3+CD4+CD20+ | CD20+ T cell |
|  | CD3+CD4+IFN-γ+ | TH1 cell |
|  | CD3+CD4+IL-17+ | TH17 cell |
|  | CD3+CD4+IL-22 | TH22 cell |
|  | CD3+CD4+CD196high +  IFN-γ/IL-22/TNF-α/GM-CSF | TH17-like cells or non-classical TH17 cells |
|  | CD3+CD8+ | Cytotoxic T (cT) cell |
|  | CD3+CD8+CD45RA+ | Naïve cT cell |
|  | CD3+CD8+CD45RO+ | Memory cT cell |
|  | CD3+CD8+CD45RO+CCR7+ | Central memory cT cell |
|  | CD3+CD8+CD45RO+CCR7- | Effector memory cT cell |
| **Monocytes** | **CD14+** |  |
|  | CD14++CD16- | Classical monocyte |
|  | CD14+CD16+ | Intermediate monocyte |
|  | CD14lowCD16++ | Non-classical monocyte |
| **NK cells** | **CD56+CD3-** |  |
|  | CD56brightCD16low | Regulatory NK cell |
| **NKT cells** | **CD3+CD56+** |  |
| **B cells** | **CD19+CD3-** |  |
|  | CD19+CD10+ | Immature B cell |
|  | CD19+CD27- | Naïve B cell |
|  | CD19+CD27+ | Memory B cell |
|  | CD19+CD27+IgD+ | Unswitched memory B cell |
|  | CD19+CD27+IgD-IgM- | Class-switched memory B cell |
|  | CD19+CD27+IgM+IgD- | IgM-only memory B cell |
|  | CD19+CD24++CD28++ | Regulatory B cell |
|  | CD19+CD5+ | Regulatory B cell |
|  | CD19+CD138+ | Plasmablast |
|  | CD19+CD38++CD10- | Plasmablast |
|  | CD19-CD3-CD138+ | Plasma cell |
